# Sunscreen and Photoprotection Habits for Patients With Porphyria and Non‐Porphyric Photosensitivity Conditions

**DOI:** 10.1111/phpp.70034

**Published:** 2025-06-23

**Authors:** David Bajek, Robert Dawe, Ewan Eadie, Victoria A. McGuire, Sally Ibbotson

**Affiliations:** ^1^ School of Medicine University of Dundee Dundee UK; ^2^ Scottish Photobiology Service, Photobiology Unit NHS Tayside, Ninewells Hospital Dundee UK; ^3^ Scottish Cutaneous Porphyria Service, Photobiology Unit NHS Tayside, Ninewells Hospital Dundee UK

**Keywords:** photoprotection, photosensitivity, porphyria, sun‐protection‐factor, sunscreen

## Abstract

**Background/Objectives:**

Individuals with photosensitivity diseases, including porphyria, face significant challenges in managing their condition due to heightened sensitivity to ultraviolet (UV) and visible light. Comprehensive photoprotection strategies are essential and prioritize environmental modifications, behavioral adjustments, protective clothing, and hats. Sunscreens serve as a supplementary measure, particularly for exposed skin areas not otherwise protected. However, the extensive variety of available sunscreen products and limited guidance on their efficacy complicate the selection process. This study evaluates sunscreen use among photosensitive individuals and gathers feedback to inform future service development and research.

**Methods:**

A questionnaire was completed by 32 individuals with porphyria (mostly with porphyrias causing skin photosensitivity) and 50 individuals with non‐porphyric photosensitivity conditions, totaling 82 participants. The survey assessed preferences and experiences with sunscreens, focusing on criteria such as protection efficacy, cost, availability, and ease of use. Responses were analyzed to identify trends and challenges in integrating sunscreens into broader photoprotection strategies.

**Results:**

Protection efficacy was the primary factor influencing sunscreen choice among photosensitive patients, highlighting its importance alongside other photoprotection measures. The survey revealed considerable variation in sunscreen use. La Roche‐Posay was the most favored brand. While 56% of porphyria patients used sunscreens explicitly labelled for visible light protection, only 11% of non‐porphyric patients did so. Overall, 40% of participants reported issues with sunscreens, and approximately half expressed dissatisfaction with certain products, emphasizing the need for more effective and user‐friendly options.

**Conclusions:**

Sunscreens are a critical adjunct to broader photoprotection strategies for photosensitive patients but are not a standalone solution. Particularly for those with porphyrias, effectiveness was regarded as especially important. The variability in product preferences and the frequent difficulties reported highlight the need for improved guidance and accessibility of sunscreens tailored to diverse photosensitivity needs. Future research should focus on developing clear recommendations and expanding options to ensure effective and personalized photoprotection.

## 1 Introduction

Cutaneous photosensitivity describes an abnormal skin response to sunlight, often triggered by ultraviolet (UV) or visible light. Conditions such as polymorphic light eruption (PLE), chronic actinic dermatosis (CAD), and solar urticaria (SU) affect many people in the UK, with PLE alone impacting up to 20% of the population [1]. These diseases range widely in severity and can severely affect quality of life.

Management strategies for photosensitive individuals typically include sun avoidance, protective clothing, and the use of sunscreens [2]. Historically, sunscreens have been targeted to protect against ultraviolet B to prevent sunburns. However, different photosensitive conditions are caused by sensitivities to different parts of the electromagnetic spectrum. For example, CAD is typically triggered by UV light and occasionally visible light, with sensitivity usually spanning UVB and UVA wavelengths (290–400 nm). Individuals with SU, on the other hand, are generally sensitive to UVA and visible light (320–700 nm). In porphyria, the accumulation of light‐sensitive compounds (porphyrins) in the skin leads to severe reactions when exposed to UVA *and* visible light, with peak absorption at approximately 400 nm, which is the boundary between UVA and visible light. As porphyrins react to both visible and UVA light, this leads to more complex and challenging photoprotection requirements for patients with porphyrias, compared with those conditions in which photosensitivity is restricted to UV wavelengths. To further complicate this variability, there remains a general underlying wavelength‐dependent sensitivity. Specifically, erythemal sensitivity to UVB is typically greater than that of UVA sensitivity, often by a factor of 10^3^ to 10^4^ (1000‐ to 10,000‐fold) [3]. As a result, more sunscreens are becoming commercially available which cover the visible range [4, 5, 6, 7], though varying coverage has often led to individuals needing to combine these with a UV protective sunscreen [8].

With hundreds of options on the market, ranging in price and efficacy, patients often face confusion regarding which products will provide adequate protection against specific wavelengths of UV and visible light, which may be an issue for patients with a range of photosensitivity conditions, not limited to the cutaneous porphyrias.

To understand the challenges faced by patients with photosensitivity, we conducted a questionnaire survey evaluating sunscreen preferences, experiences, and application habits. The survey explored factors like cost, availability, and ease of use to identify trends and challenges in incorporating sunscreens into photoprotection strategies. These insights aim to improve guidance and resources, enhancing patients' quality of life and safety in sun exposure.

## 2 Materials and Methods

### 2.1 Questionnaire Design

A structured questionnaire (Supporting Information) was developed to assess the preferences and experiences of sunscreen usage among individuals with photosensitivity conditions. The questionnaire was primarily designed based on our in‐clinical discussions and insights gathered from patient‐engagement events. It aimed to better understand the factors influencing sunscreen choice, such as important criteria, brand preferences, and usage habits, to ultimately improve our ability to support patients' photoprotection needs.

Participants were asked to respond to questions regarding their criteria for selecting sunscreens (SPF, formulation for example), perceived efficacy of the products they used, and their habitual application practices. Additionally, the questionnaire explored various determinants that might influence their choices, including cost, product availability, and user‐friendliness.

The first question in the survey aimed to establish the range of diagnoses experienced by the respondents. To ascertain the importance of various photoprotection methods, participants were asked to rate the importance of sunscreen, glasses/eye protection, clothing/hats/skin coverings, sun‐avoidance, and UV‐filters on windows. Ratings ranged from 0 = not important to 4 = very important. Respondents were then asked to provide the reasons why they use their chosen sunscreen(s), with the option to select multiple answers. Subsequent questions asked whether participants had experienced any problems or side effects from using sunscreen, which aspects they disliked the most, and whether they experienced any non‐skin symptoms (e.g., fatigue or headache) during or shortly after photosensitivity flares—and whether any sunscreens had reduced or prevented these.

### 2.2 Implementation and Delivery

The questionnaire was distributed electronically over the timeframe of April 2024 to August 2024. The electronic format was chosen to facilitate efficient distribution, completion, and data collection. While a paper‐based option may have enhanced accessibility for some individuals, the electronic approach was more practical for reaching a wider audience within the available networks and allowed for streamlined analysis.

### 2.3 Population Groups Targeted

Two distinct distribution pathways were used. For the porphyria group, the questionnaire was distributed through the British Porphyria Association (BPA), who shared it via their newsletter and social media channels with the message: “Do you have one of the skin porphyrias and a few minutes to spare?” This national patient support organization was best positioned to reach individuals with photosensitive cutaneous porphyrias across the UK. For the non‐porphyric group, the questionnaire was sent to 124 patients of the Scottish Photobiology Service (SPS), all of whom had provided prior consent to be contacted for service evaluation and quality improvement projects. The use of different distribution channels reflected the distinct population access points available for each group.

### 2.4 Statistical Analysis

This was a semi‐quantitative descriptive survey. No power calculation was feasible or undertaken, as the aim was to collect as many responses as practically possible, and no formal statistical comparisons were intended between groups. Responses were summarized using frequency counts, and where applicable (e.g., photoprotection ratings), group averages were calculated. No inferential statistical testing was performed. Responses were first presented separately for each group and then combined to provide an overview of trends and shared challenges across all participants.

### 2.5 Ethical Considerations

This project was registered with the Clinical Governance team at NHS Tayside, who confirmed that ethical approval was not required.

## 3 Results and Discussion

The questionnaire was responded to by 32 individuals with porphyria (mainly cutaneous and photosensitive) and 50 with non‐porphyric photosensitivity conditions (82 participants in total). No demographic information (gender, age) was collected by the questionnaire.

Regarding the range of diagnoses experienced by the questionnaire responders, most of those in the porphyria group (84%) specified a diagnosis of Erythropoietic/X‐linked Protoporphyria. Aside from 13% unstated, the remaining 3% were individuals with Porphyria Cutanea Tarda. In the non‐porphyric photosensitivity group, 34% elected not to disclose a particular diagnosis, giving the majority of those reported as SU (24%), PLE (16%), and CAD (14%).

**FIGURE 1:**
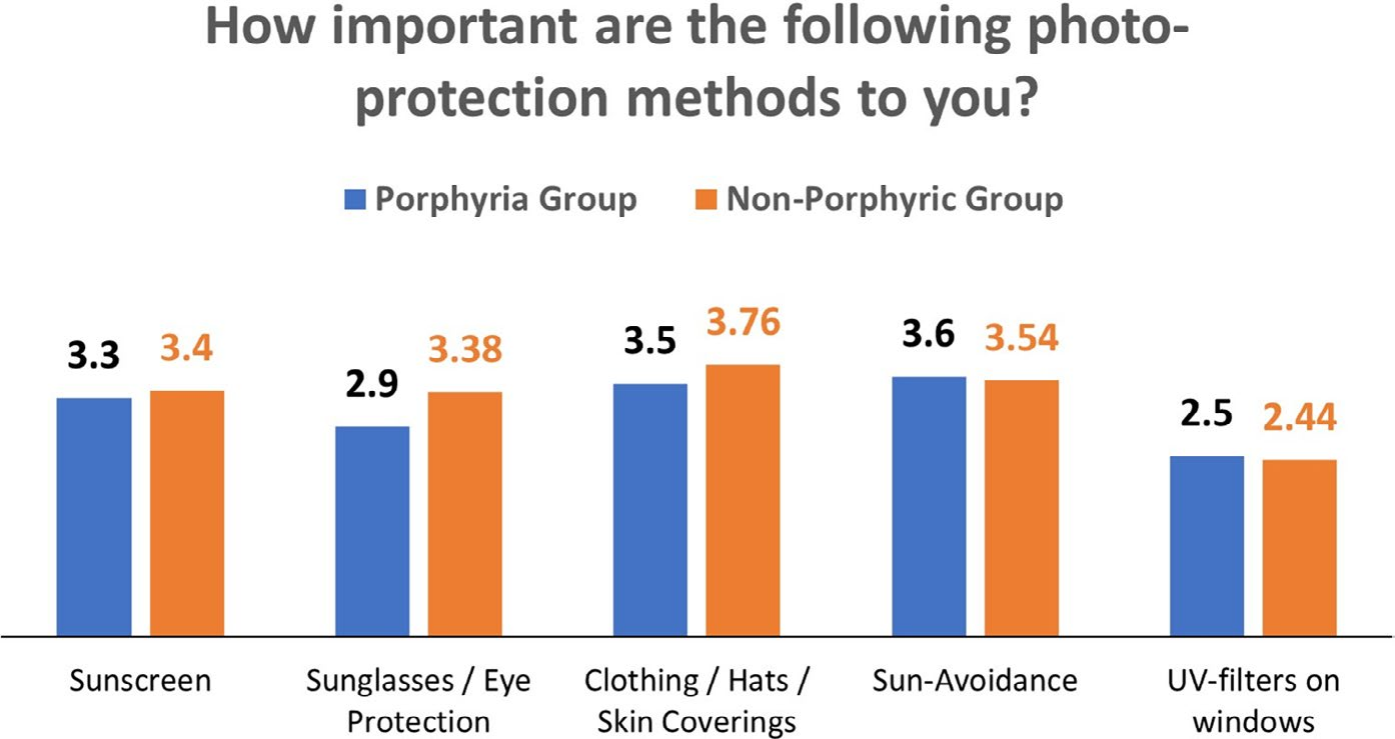
Individual's average responses to rating the importance of various photoprotection methods.

Regarding the importance of various photoprotection methods, scores were assessed and then averaged for each group (Figure 1).

On average, both groups rated each method of photoprotection as more than ‘moderately important’ (i.e., to say, a score of at least 2), with sunscreens, clothing and sun‐avoidance all rating more than ‘important’ (i.e., a score of 3). Both groups rated each method similarly with no significant differences between them. The least important method was the use of UV‐filters on windows.

**FIGURE 2:**
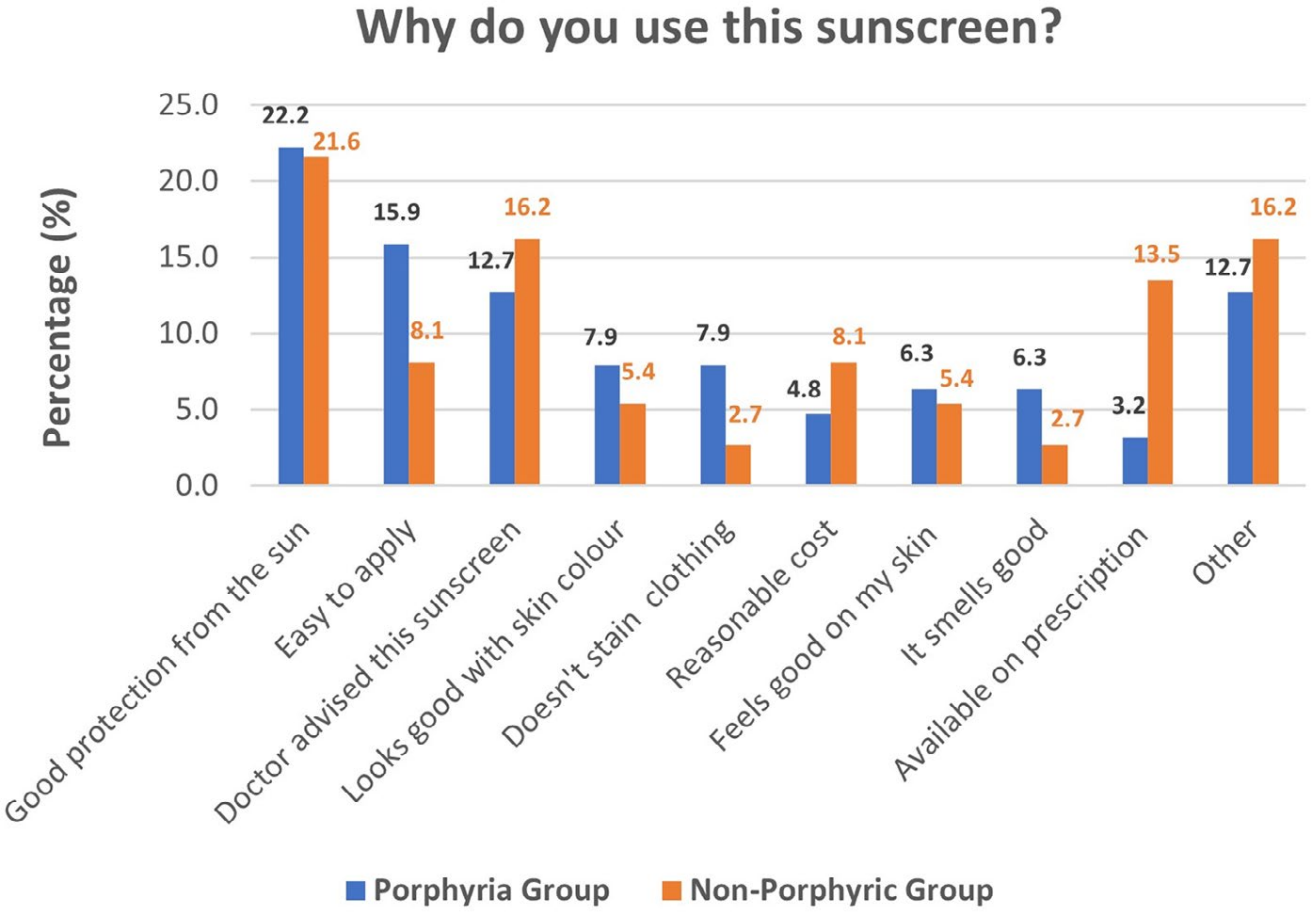
The reasons provided by individuals for their choice in sunscreen, above as absolute values, below as percentages of each group with respect to the total responses.

Regarding their reasons for using their sunscreen of choice, the groups responded as follows: see Figure 2.

The response “Gives me good protection from the sun” is the most cited reason for using a specific sunscreen, especially among the porphyria group, with 22% of respondents providing this reason. The non‐porphyric photosensitivity group also values sun protection, but the percentage is noticeably lower than that of the porphyria group, suggesting other factors play a more significant role in their sunscreen choice. “Ease of application” is more favoured by the porphyria group; likely this is due to the visible portion of their sensitivity requiring a pigmented sunscreen, which must ideally aesthetically suit their complexion.

Though the question was not directly asked in the questionnaire, when providing details of sunscreens, the porphyria group individuals were more likely than those in the non‐porphyric photosensitive group to voluntarily indicate whether the specific version of their brand of sunscreen would protect against visible light. We found that 56% of the porphyria group were using visible‐protection sunscreens, 32% were not, and 12% could not be determined. On the other hand, 11% of the non‐porphyric photosensitive group were using visible‐protection sunscreens, compared to 57% who were not, with 31% undetermined. Visible light sensitivity may be less of an issue for individuals in the non‐porphyric group, and therefore, as a group, they may have less reason to be aware of visible‐light‐protecting sunscreens. The porphyria group also appears more proactive about using sunscreens that protect against visible wavelengths, which aligns with their specific needs for photoprotection to these wavelengths. This analysis suggests a need for better awareness and accessibility of sunscreens that protect against both UV *and* visible wavelengths to ensure effective management of their condition.

**FIGURE 3:**
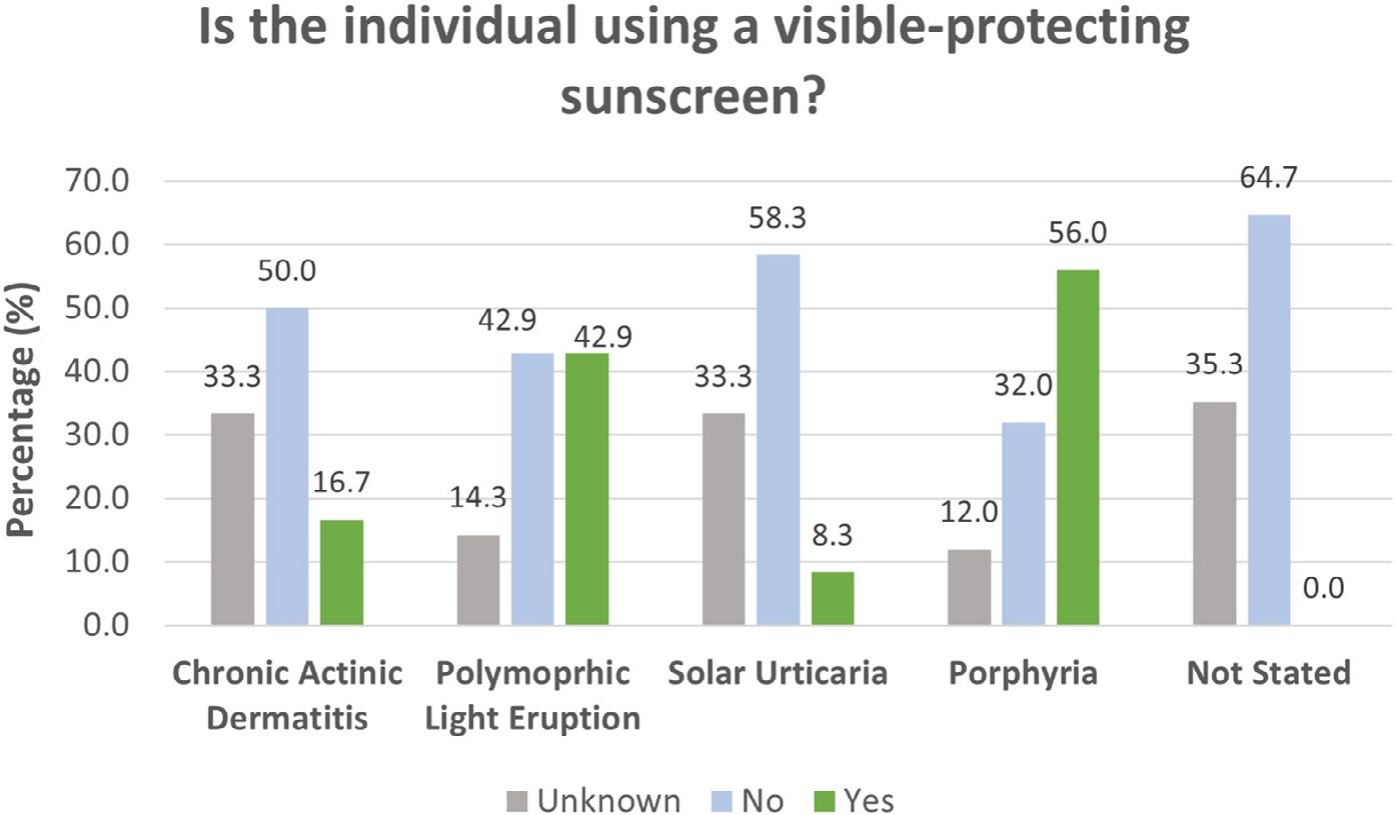
Determining which individuals (with which diagnosis) were currently using visible‐protection sunscreens.

The analysis of whether individuals are using visible‐protecting sunscreen reveals condition‐specific trends; see Figure 3.

Use of visible‐light‐protective sunscreens varied by diagnosis. Uptake was highest among individuals with porphyria (56%), as expected, but notably low in conditions like solar urticaria (8.3%) and among those without a stated diagnosis (0%). Moderate use was seen in PLE (42.9%) and CAD (16.7%), despite differing relevance of visible light in these conditions. These differences suggest gaps in awareness or access to appropriate sunscreen types. We then assessed responses to the question ‘Have you ever had any problems or side effects from using any sunscreen’. In the porphyria group, 28.6% reported “Yes”, in contrast, with 53.2% of the non‐porphyric photosensitivity group. One possible explanation might be that the non‐porphyric photosensitive group may be more prone to experiencing adverse effects from sunscreen use compared to the porphyria group, potentially due to differences in their immune‐based photosensitivities, product selection or indeed reporting habits. For example, we note that over 75% of patients with CAD will have allergies to various agents [9], where distinction (for patients) between allergic and photosensitive reactions may be difficult.

**FIGURE 4:**
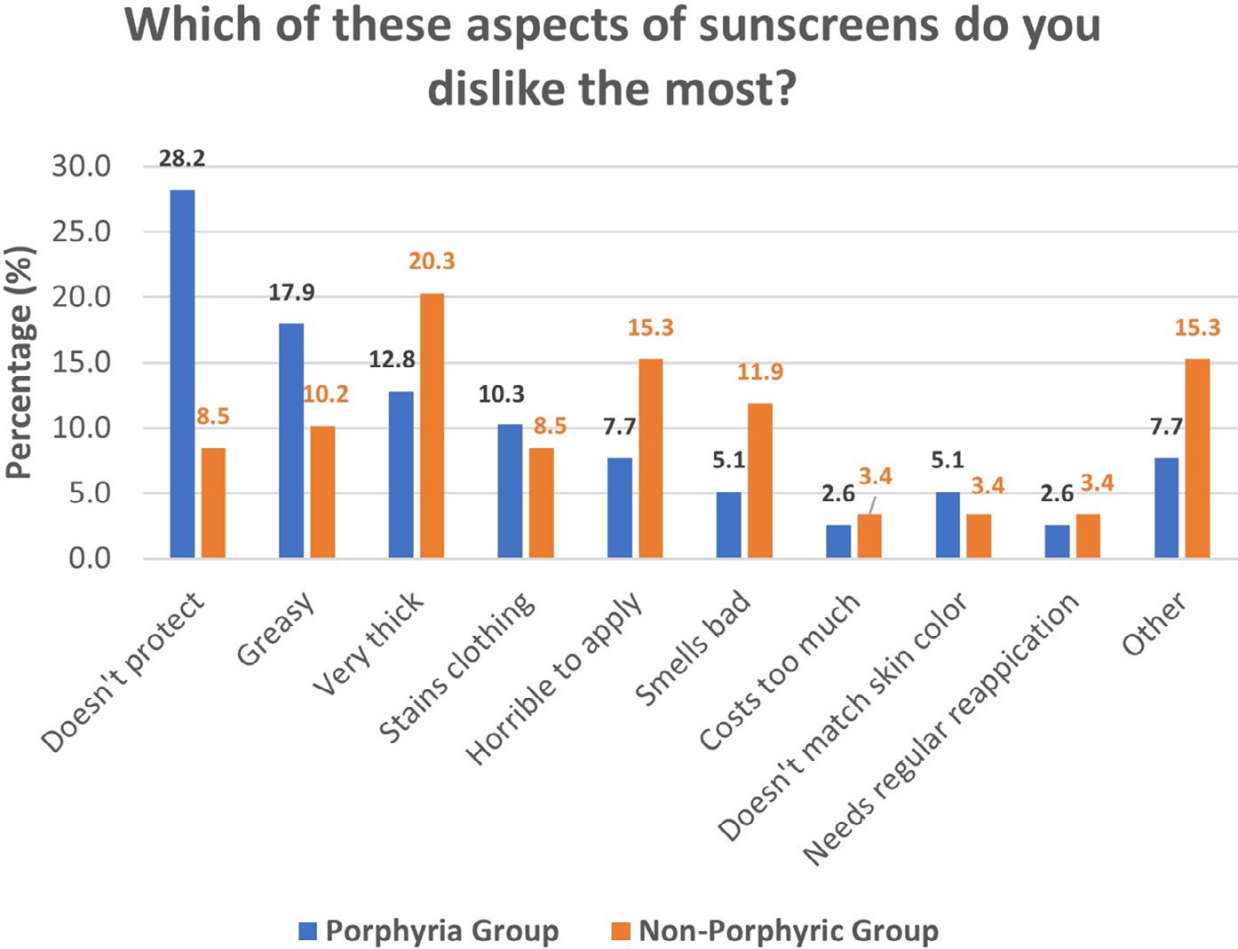
Summary of the noted dislikes of sunscreens across both groups.

Approximately half of the entire surveyed group (porphyria group, 53.6%, non‐porphyric photosensitivity group, 48.9%) reported that there were sunscreens which they did not like and so would not use. These sunscreens ranged in brand and type, and so no other conclusion is drawn other than to reinforce that a large proportion of the responders with photosensitivity struggle, or have struggled, with sunscreen selection. When asked which aspects of sunscreens individuals disliked the most, the responses varied across both groups (Figure 4).

Lack of protection rated highest within the porphyria group, at 28.2% compared to just 8.5% of the non‐porphyric photosensitivity group, who most disliked sunscreens which were very thick (20.3% compared with just 12.8% of the porphyria group). The contrast in results suggests that those diagnosed with porphyria have more dislikes associated with sunscreens than the non‐porphyric photosensitivity group, who appear to have relatively balanced dislikes in sunscreens. We consider these findings highlight that light protection is of primary importance for the porphyria group. Given the limited availability of sunscreens that effectively protect against visible light, and the lack of information on the packaging about visible light protection, these patients are likely to face greater challenges in identifying suitable products. In contrast, other photosensitivity conditions are often mediated primarily by UVA and UVB radiation, for which a wider range of appropriate sunscreens is available. These increased options allow non‐porphyric patients with photosensitivity to consider additional factors such as scent, application texture, and cosmetic acceptability when choosing a sunscreen. For porphyria patients, however, the scarcity of options means such considerations are secondary, as their choices are dictated predominantly by the need for adequate light protection.

**FIGURE 5:**
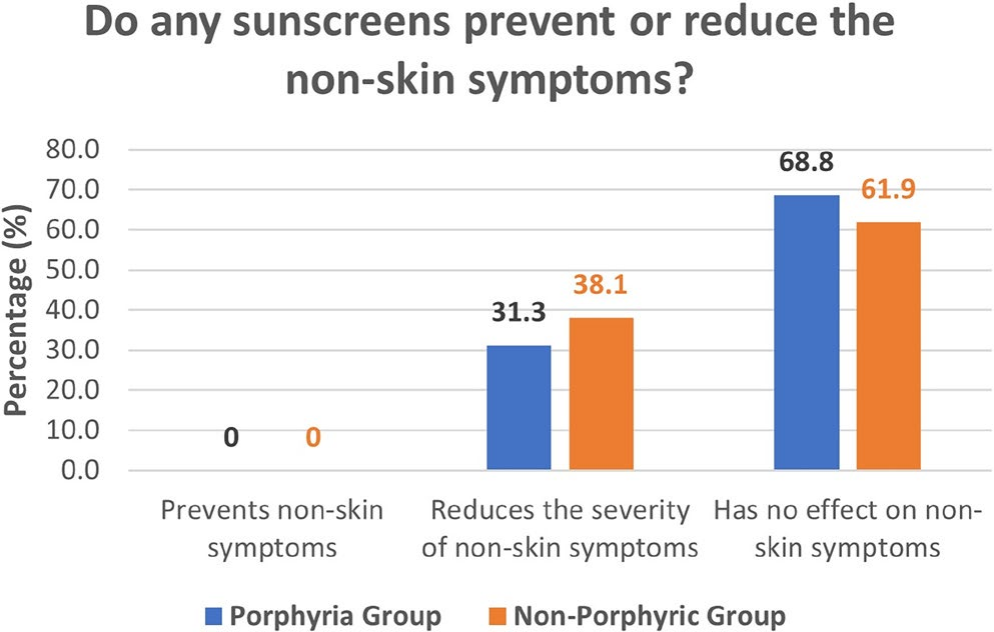
Group response to whether any sunscreens prevent, reduce or have no effect on non‐skin symptoms.

We enquired whether individuals experienced any non‐skin symptoms during or shortly after photosensitivity flares (such as fatigue or headache). Regarding whether individuals experienced non‐skin symptoms, approximately half of the entire group reported “Yes”; 57.6% of the porphyria group and 44% of the non‐porphyric photosensitive group. On asking whether any sunscreens prevent, reduce, or have no effect on these non‐skin symptoms, the results are as follows, see Figure 5.

No individual reported prevention of systemic symptoms in either group, whilst only approximately a third of the combined group population reported a reduction in symptoms (31.25% of the porphyria group, 38.1% of the non‐porphyric photosensitive group). The remaining majority (approximately 65% of the combined population) reported that sunscreen had no effect on non‐skin symptoms.

Limitations of this study include the lack of demographic data such as age, gender, skin phototype, or clinical setting, which restricted subgroup analysis. Skin type may influence sunscreen behaviour independently of photosensitivity diagnosis, with individuals with darker skin potentially adopting different habits or levels of reliance on sunscreen. The low number of respondents with Porphyria Cutanea Tarda (PCT), which typically presents in mid‐life as an episodic condition, likely reflects lower engagement with patient support groups at the time of survey dissemination [10, 11]. This contrasts with individuals diagnosed in childhood with protoporphyrias (e.g., EPP or XLP), who often experience lifelong, daily light sensitivity and may be more consistently engaged with organisations like the BPA. Additionally, reliance on self‐reported responses introduces the potential for recall or reporting bias.

## 4 Conclusions

This study highlights the variability in sunscreen use among individuals with photosensitivity, particularly the distinct needs of those with porphyria who prioritize visible light protection. Sunscreens remain a key but supplementary element of broader photoprotection strategies. The findings point to gaps in guidance, product accessibility, and formulation suitability, underscoring a need for clearer patient education and more inclusive product development. Addressing these challenges will require coordinated efforts between clinicians, researchers, and manufacturers to ensure sunscreen options are tailored to specific conditions and patient preferences. Future research should evaluate product efficacy more broadly and explore ways to personalize photoprotection advice.

## Author Contributions

Conceptualization: Ewan Eadie and David Bajek. Methodology: Ewan Eadie and David Bajek. Formal analysis: David Bajek, Victoria A. McGuire. Investigation: All. Data curation: David Bajek, Victoria A. McGuire. Writing – original draft preparation: David Bajek. Writing – review and editing: All. Supervision: Ewan Eadie and Sally Ibbotson. All authors have read and agreed to the published version of the manuscript.

## Ethics Statement

This project was registered with NHS Tayside's Clinical Governance department, project registration number 37/24. Ethical review and approval were not required for this survey.

## Consent

The authors have nothing to report.

## Conflicts of Interest

S.I. declares that, during the period of 2024, she received financial support from L'Oreal/La Roche Posay for her costs in attending the Skin Alliance Forum in Paris in October 2024, and the Photobiology Unit will receive a financial contribution in support of the Introduction to Photodermatology Course, Dundee, scheduled for April 2025.

## Supporting information


Data S1.

## Data Availability

Data from this publication can be sought from the corresponding authors upon reasonable request.
