# Supplementary material for: Sunscreen and Photoprotection Habits for Patients With Porphyria and Non‐Porphyric Photosensitivity Conditions
Source: Photodermatol Photoimmunol Photomed. 2025 Jun 23;41(4):e70034. doi: 10.1111/phpp.70034 (PMC12183616; doi:10.1111/phpp.70034)
Supplement: Supplementary file 1 — Data S1. [file PHPP-41-e70034-s001.pdf]

Sunscreen Questionnaire

The Scottish Photobiology Service would like to gather information on the sunscreens you use, the sunscreens you like and the reasons why. Please help us by completing this short questionnaire.

* Required

1. Are you diagnosed with any photosensitivities? *
   - ◯ Yes
   - ◯ No
   - ◯ Prefer not to say

2. If you selected 'Yes' to the previous question, we would appreciate details of your light sensitivity diagnosis below. This question can be skipped if you prefer.

3. Do you use sunscreen? *
   - ◯ Yes
   - ◯ No

4. If you selected 'No' to the previous question, please detail the reasons why.

5. Do you use more than one sunscreen at a time?
   If yes, please answer the remaining questions as if referring to both sunscreens where possible. *
   - ◯ Yes
   - ◯ No

6. What is the name of the sunscreen(s) you currently use? Please give as much detail as possible. *

7. Why do you use this/these sunscreen(s)? (select as many answers as you like) *
   - ☐ It works well with my skin colour / it looks good
   - ☐ It is easy to apply
   - ☐ Doctor advised this sunscreen
   - ☐ It is available free on prescription
   - ☐ It feels good on my skin
   - ☐ It smells good
   - ☐ The cost is reasonable
   - ☐ It doesn't stain my clothing
   - ☐ It gives me good protection from the sun
   - ☐ Other

8. Is the sunscreen you use also your favourite sunscreen? *
   - ◯ Yes
   - ◯ No

9. If you answered No to the previous question, what is your favourite sunscreen? Please give as much detail as possible. *

10. If you are not using your favourite sunscreen, why not?

11. Why is it your favourite sunscreen? *

- [ ] The cost is reasonable
- [ ] It gives me good protection from the sun
- [ ] It smells good
- [ ] It doesn't stain my clothing
- [ ] It works well with my skin
- [ ] It is easy to apply
- [ ] It is available free on prescription
- [ ] It feels good on my skin
- [ ] Other

12. Please score the following options in terms of importance for you when choosing a sunscreen
0 = Not important
1 = Slightly important
2 = Moderately important
3 = Important
4 = Very Important *

	0	1	2	3	4
Cost	○	○	○	○	○
Easy to Apply	○	○	○	○	○
Feels Good	○	○	○	○	○
Smells Good	○	○	○	○	○
Doesn't Stain Clothing	○	○	○	○	○
Protection from Sun	○	○	○	○	○
Looks Good	○	○	○	○	○
Protects for a long time / less need to reapply	○	○	○	○	○
Available on Prescription	○	○	○	○	○

13. Have any other sunscreens provided you with better protection than the sunscreen you currently use? *

- ○ Yes
- ○ No

14. Which sunscreen provided you with better protection? Please give as much detail as possible. *

15. Why don't you use the sunscreen that provides better protection? *

16. Have you ever had any problems or side effects from using any sunscreen? *

- ○ Yes
- ○ No
- ○ Not applicable / Don't use sunscreen

17. Which sunscreen did you have problems or side effects from? Please give as much detail as possible.

18. What problems did you have?

19. Are there any sunscreens you don't like? *

- ○ Yes
- ○ No

20. Which sunscreen don't you like? Please give as much detail as possible.

21. Why don't you like this sunscreen? (select as many options as you wish) *

- [ ] Horrible to apply
- [ ] Very thick
- [ ] Doesn't protect me
- [ ] Stains clothing
- [ ] Greasy
- [ ] Need to reapply regularly
- [ ] Doesn't work with my skin colour
- [ ] Costs too much
- [ ] Smells bad
- [ ] Other

22. Do you have non-skin symptoms during, or shortly after, your photosensitivity flares (for example fatigue, migraines etc.)? *

- ○ Yes
- ○ No

23. Do any sunscreens prevent or reduce the non-skin symptoms? *

- ○ Prevents non-skin symptoms
- ○ Not applicable / don't use sunscreen
- ○ Has no effect on non-skin symptoms
- ○ Reduces the severity of non-skin symptoms

24. How important are the following photo-protection methods to you?
0 = Not important
1 = Slightly important
2 = Moderately important
3 = Important
4 = Very Important *

	0	1	2	3	4
Sunscreen	○	○	○	○	○
Sunglasses / Eye Protection	○	○	○	○	○
Clothing / Hats / Skin Coverings	○	○	○	○	○
Sun-Avoidance (for example, seeking shade)	○	○	○	○	○
UV-filters on windows	○	○	○	○	○

25. If not listed in the previous question, which other photo-protection methods do you use, and how would you rate it's importance. Please list the protection method, followed by its importance to you. For example, sunglasses - 2.
0 = Not important
1 = Slightly important
2 = Moderately important
3 = Important
4 = Very Important

26. If you have any other comments or information you would like to make us aware of, please detail below.

This content is neither created nor endorsed by Microsoft. The data you submit will be sent to the form owner.

Microsoft Forms
